# British Medical Association: Proceedings of Sections

**Published:** 1895-08-10

**Authors:** 


### British Medical Association.

### PROCEEDINGS OF SECTIONS.

### Medicine.

In opening the proceedings Dr. Pavy pointed out the great advances which had been made owing to the discovery of the relationship existing between the growth of certain micro-organisms and the occurrence of certain diseases. He urged also that the influence which this knowledge would have upon treatment might prove very great indeed, and that it might, in fact, enable us to exercise considerable control over the spread of infectious diseases.

Dr. Sidney Martin introduced a discussion on diphtheria and its treatment by antitoxin. This method of treatment was not merely empirical, but was the result of most careful study of the pathology of the disease. It had been possible to isolate the toxin to which the symptoms were largely due, and by a series of diagrams he showed the effects of introducing this toxin into rabbits. The temperature became irregular, and wasting, paralysis, and death took place. Having then described the nature and production of the substance spoken of as antitoxin, he showed that the injection of this material alone into the veins caused but little change of temperature of the body and no loss of weight. It seemed, in fact, to be harmless. Little effect, however, as it had upon the bodies of healthy animals, it had great influence in enabling them to resist the action of the toxin of diphtheria, for on the intravenous injection of toxin and antitoxin at the same time only slight symptoms of poisoning by the toxin occurred, a slight neive degeneration being the only injurious effect.

It was also pointed out that injection by antitoxin seemed to protect animals from the occurrence of fatty degeneration of the heart under the influence of diphtheria poison, and that it was therefore to be expected that with the extension of the antitoxin treatment there would be fewer cases of death from heart failure than had been the case before.

Dr. Goodall gave some details of his clinical experience in the use of the remedy, the use of which he considered the best treatment at present available.

Professor von Ranke, of Munich, strongly supported the treatment by serum. In regard to death-rates, whilst in 1892 he had a hospital mortality of 56·2 per cent., in 1893 of 46 per cent., and in 1894, up to September 24th, when he commenced the serum treatment, of 57 per cent., his death-rate had since that date been reduced to 17·7 per cent. He thought also that clinically, putting statistics aside, the course of the disease in the individual cases was markedly influenced for good.

Mr. Lennox Browne did not agree with some who had spoken in favour of the serum treatment.

Professor Baginsky, of Berlin, stated that whilst the mortality in the four years previous to 1895 had been, on the average, under the old system of treatment, 41 per cent., it had during the last year, under the serum treatment, been reduced to 15·6 per cent.

Dr. Johnston, of Glasgow, Dr. Hermann Biggs, of New York, Mr. Campbell Hall, and Dr. Sims Woodhead also spoke, the latter urging the advantage of using large doses of serum, and relating statistics from Paris favourable to the treatment.

Dr. Samuel West read a paper on the "Treatment of Diabetes Mellitus by Uranium Nitrate," a drug which he considered the most powerful we possessed in the treatment of this disease.

A discussion on acute lobar or croupous pneumonia was introduced by Dr. Douglas Powell. Notwithstanding improved public health in regard to other diseases, especially those of the zymotic class, no practical abatement was to be noted in the mortality from pneumonia. In regard to its etiology, he insisted on the importance of other factors besides the occurrence of micro-organisms, and pointed out the relationship of the disease to severity of climate and especially to lowness and changeableness of temperature. It had also to be remembered that Fraenkel's pneumococcus had been found in healthy saliva, in the exudation in meningitis, in ulcerative endocarditis, and in otitis media, diseases which had little pathological resemblance to pneumonia, and with which pneumonia was not commonly associated. In regard to treatment, he came to the conclusion that the ordinary saline drug treatment was based upon sound principles in uncomplicated cases, whilst when there were special indications for their use, strychnine, caffeine, digitalis, and morphine were most valuable remedies. As to the proposed treatment by antitoxin, he was still of open mind; he had not had an opportunity of trying it, but he thought it would probably be along these lines that progress would be made in the future. Dr. Washbourn insisted upon the great importance of revising our nomenclature of the subject, and speaking of pneumococcal meningitis, pleurisy, &c., in all those cases in which Fraenkel's coccus could be demonstrated.

Dr. Pollock considered that bacteriological diagnosis was over-rated in importance, while Dr. Tyson thought that bacteriology explained the great differences clinically met with in various cases. Dr. Baümler, of Freiburg, insisted on the importance of cold bathing in the treatment of pneumonia. Many other speakers joined in the discussion.

### Surgery.

The proceedings of this section commenced by the delivery of an address by its President, Sir William MacCormac, on "Some Points of Interest in connection with the Surgery of War." He drew attention to the great changes produced by the introduction of the modern rifle sending a small bullet with a great range and an enormous velocity. The range was stated to be about 3,500 yards, a little over two English miles, the trajectory flatter, and the direction more true, results attributable to the diminished resistance of the air and the greater driving power of the new powder.

The factors determining the amount of damage inflicted by a gunshot wound were the unalterable form of the projectile, as well as its size, the velocity on impact, and the resistance of the tissues. About nine-tenths of the casualties on a modern battlefield were due to rifle bullets, and with the use of magazine rifles firing sixty shots a minute and carrying immense distances, it was not likely that this proportion would diminish.

A full account was given of the experiments which had been made on dead bodies to ascertain the character of the injuries actually produced by these new projectiles, and emphasis was laid on the so-called explosive effects caused by them, especially in traversing closed cavities. This rending process appeared to be due to rapid arrest of the flight of the bullet on traversing fluid or semi-fluid matter, and the consequent distribution of its energy over a large area, causing disruption of the walls of the cavity, the bones of the skull being separated and split open, and terrible injuries being inflicted upon semi-fluid organs, such as the liver, spleen, a full stomach, or intestine. Where bones were perforated also great injury was produced; not only were the shafts of bones broken up into small pieces, but the soft parts beyond the bones were greatly lacerated, the adjacent muscles being filled with *débris* and detached fragments of bone, and the exit wound being large and ragged.

As regards the penetrating power of the bullets, they were found in nearly every case to go straight through the part struck, and the old-fashioned contour shot was never met with. The human body was traversed with ease at 2,000 yards, and the skull, thorax, and abdomen would suffer alike. Up to this distance the bullets went clean through the body, unchecked even by the hardest bone. The knowledge that this was so would render unnecessary that disastrous searching for lodged bullets which formed so considerable a part of the older military surgery—a search which often involved more risks than any the bullet would produce. It was, of course, necessary to remove such portions of clothing as were carried in, as they were septic, but fortunately this incarrying of clothing was much less common with the small bullets than it was when large ones were used. At longer ranges the flesh wounds produced were comparatively simple, but it was not to be forgotten that in actual warfare a large number of wounds were caused by bullets which, in consequence of having already struck the ground or a wall, had become deformed, and in these cases the laceration even at the point of entry might be very great.

In conclusion, it might be stated that with magazine quick-firing rifles it would be impossible to maintain a contest at short range, and that probably the bulk of the gunshot wounds of the future would be produced at long distances, and, from the narrow bullet track, the small external openings, often approaching the subcutaneous in character, and the moderate degree of comminution and fissuring of the bone, will be capable of successful surgical treatment.

A discussion on "The Diagnosis and Treatment of Fractures of the Upper Third of the Femur, including the Neck," was introduced by Sir William Stokes, M.D., with a lantern demonstration. He endorsed the view that the angle between the neck and the shaft was normally unaffected by age, and that osseous union might take place in several forms of fracture of the cervix femoris.

Sir George Humphrey considered that a fracture in any part of the skeleton would unite if the parts were kept in apposition at whatever time of life it occurred. Mr. Bryant, Mr. Mayo Robson, Mr. Greig Smith, and others, thought that in young subjects impaction of the fracture should be reduced so as to obviate deformity.

Surgeon-Colonel Keegan gave a demonstration on "Rhinoplasty in India," and Dr. Kendal Franks read a paper on "Movable Kidney." Mr. Howard Marsh read a paper on "Some Rare Forms of Bony Ankylosis." It was not true as held by some that bony ankylosis only occurred as the result of suppuration, nor that suppuration invariably led to it if recovery took place. In pyæmia, in acute suppurative arthritis following wounds, and in many chronic tuberculous diseases of joints recovery might supervene without ankylosis, and in osteo-arthritis, tuberculous joint disease, and gout, and other cases bony ankylosis might take place, although no suppuration had occurred. Among such cases he mentioned those of ossification of the intervertebral discs throughout the whole spinal column.

Mr. Buckston Browne read a paper describing how a vesical calculus impacted between the lateral lobes of an enlarged prostate and the wall of the bladder might be incapable of being either reached by the sound or crushed by the lithotrite, and would therefore require to be removed through a suprapubic incision.

Mr. Butlin introduced a discussion on the "Surgery of Tumours of the Thyroid Gland."

### Obstetrics and Gynæcology.

In opening the proceedings of this section Sir William Priestley gave an address on "Over-operating in Gynæcology." After referring to the influence exercised on the obstetric art by the introduction of antiseptics, anæsthetics, and improvements in the construction of the instruments, he said that it was in the department of gynæcology that the greatest activity was to be noted in these later days. The success that had been attained in operative work was such as to evoke unqualified admiration, and to persuade some people that henceforth there need be no limits prescribed to exploits in abdominal surgery, but the enthusiasm aroused, not without good reason, might readily be carried too far. He had lived long enough to have seen the evil of rushing on too impetuously, and in watching the progress of gynæcology had witnessed the wax and wane of many enthusiasms which had had a share in bringing something like discredit on a department of practice which, rightly exercised, was productive of great good, but, exercised unwisely, was capable of producing infinite harm. He could recollect what had been termed a craze for inflammation and ulceration of the os and cervix uteri. Shortly afterwards came a brief and not very creditable period when "clitoridectomy" was strongly advocated as a remedy for numerous ills. This fortunately, was speedily abandoned. Then followed a time in which displacement of the uterus held the field, and every backache, every pelvic discomfort, every general neurosis was attributed to mechanical causes, and must needs be treated by uterine pessaries. Again, we had an epoch when oöphorectomy or castration of women was not only recommended and largely practised as a means of restraining hæmorrhage in bleeding fibroids, but also as a remedy for certain forms of neurosis even when the ovaries were healthy or not seriously diseased. Close upon this, again, came an ardour for stitching up rents in the cervix uteri following childbirth. One votary of this practice boasted of having detected and operated on in a short period no fewer than 300 or 400 cases which he had found in examining 900 women. No such experience, so far as he knew, had been chronicled by any other author. Lastly, we had what had been described as an epidemic of operations for excision of the uterine appendages; and even now, although this operation had but recently come into vogue, there was a demand for more conservative methods which should leave these parts of the generative system a chance of still performing their important functions. In most or all the modes of treatment which he had indicated there was probably some utility, if properly limited, but the germ of truth had been obscured by inappropriate use. It seemed to him that a too reckless attempt at progress not only impaired the reputation of gynæcology but that experience was gained at the expense of much suffering to many patients—patients of the gentler sex, on whom no man with a spark of chivalrous feeling would desire to inflict unnecessary pain. The first instinct should be to try if an operation could be avoided, not to seek reasons for performing it. No amount of success in operating was of value unless it were shown that operations were necessary. Recoveries from operations did not necessarily justify them. Nor should the urgent wishes of the patients outweigh the counsels of prudence in regard to operations. There were always discontented women who magnified their sufferings, and neurotic women who would undergo any martyrdom for the sake of evoking sympathy. Serious and dangerous diseases justified serious remedies, but it was quite a different matter to submit poor women to capital operations for small or large uterine fibroids without symptoms, or symptoms not of an urgent character, to open the abdomen for the cure of uterine displacements attended only by discomfort, or to remove the ovaries for indefinite nerve pains or other subjective symptoms. In his opinion such proceedings were absolutely unjustifiable. The announcement of some new proceeding was apt to be taken up as the last new thing in science by many imitators, who used it, perhaps, in most inappropriate cases. Thus he had known the uterine appendages removed for matting together of parts by pelvic cellulitis and peritonitis after delivery, because the patient was too impatient to wait for the slow recovery which would have taken place in due course. In like manner he had heard of the ovaries being removed for chronic metrorrhagia after abortion. Curetting, because it was new in fashion, had been proposed to cure a somewhat copious loss of blood, due to the approach of the climacteric, which after a time disappeared spontaneously. The same treatment had within his experience been recommended for sterility in a young wife recently married, and who had no symptoms beyond her impatience to have children. Something operative must be done, and in lack of indication for anything else, this was proposed in all good faith. But the motto of all should be, *Primo non nocere*—at least do no harm.

### Public Medicine.

Mr. Ernest Hart delivered an address on "Public Health Legislation and the Needs of India," in which he drew attention to the wants of the country in regard to sanitary legislation, and to the grave defects in such sanitary laws as we possessed. But he especially laid himself out to show the antiquated nature of the regulations in force in regard to sanitation in India, and to pointing out the loss of life thus entailed upon the population, both military and civil. A resolution was afterwards passed by the Section, asking for the appointment of a Royal Commission or a Departmental Committee for the consideration of the points to which Mr. Hart had drawn attention.

Dr. Legge opened a discussion on "The Regulation of the Slaughter of Animals for Human Food," by a paper in which he emphasised the evils which attached to the present system. In conclusion, he said that the recent report of the Royal Commission appointed to inquire into the effect of food derived from tuberculous animals had come to very definite and startling conclusions, which could not be without effect on public opinion. Public opinion was at the present time lukewarm on the subject, and even the medical profession had not devoted the attention to the subject of the transmissibility of tubercle to man through meat or milk that it deserved. There was, too, the undeniable hostility on the part of the various meat traders' and butchers' associations to anything that was likely to drive the control of the trade by centralisation into the hands of a few large firms. But the evils of the system at present adopted in this country were so apparent that he hoped the whole question of meat inspection would before long be made the subject of a Royal Commission. In reply to several speakers, he pointed out that there was no power at present to close private slaughter-houses wholesale either in London or provincial cities or towns. London, however, had it in its power to close any private slaughterhouses whose use was objectionable or undesirable by the non-renewal of license. Provincial cities and towns could acquire the same power by the adoption of the Public Health Amendment Act. In rural districts, unless the sanitary authority had obtained urban powers, there was entire absence of control over private slaughter-houses.

On the motion of Dr. Newsholme, a resolution was passed urging the Government to appoint a Commission to enquire into the whole question of meat supply and inspection.

A discussion on "Sewer Ventilation" was opened by Mr. J. Parry Laws, and Dr. Neech related a series of interesting experiments undertaken with the object of showing the movements of air in sewers.

Dr. Waldo and Dr. Walsh dealt with underground workshops, and Brigade-Surgeon Lieutenant-Colonel Pringle with smoke abatement. A paper was then read by Dr. Boobyer on "Hospital Isolation and the Disinfection of Patients." He thought more stress should be laid upon the treatment of enteric fever on the lines of the other infectious diseases.

Dr. J. Spottiswoode Cameron agreed as to the necessity of isolating enteric fever. He regarded the comparative freedom of Huddersfield from that disease as due to the free use of the infectious diseases hospital for its isolation.

Dr. Barwise was in favour of large central hospitals as being cheaper both to administer and to build.

Dr. Caverhill was against the use of tents for the acute stages of infectious disease. Such hospitals should only be supplementary to more stable buildings.

Dr. Fosbroke thought patients could be safely removed more than five miles; but Dr. Boobyer, in reply, mentioned cases where great difficulties would be experienced in removing great numbers of patients great distances. Under such circumstances extreme centralisation would be found to be a mistaken policy. It was extremely fatal to remove typhoid cases after the second week.

Dr. Klein introduced the subject of "The Diagnostic Value of the Diphtheria Bacillus," and was followed by Dr. Hermann Biggs, whe read a paper on the same subject.

### Psychology.

The President (Dr. Mickle) opened the proceedings by an address on "Abnormal Forms and Arrangement of Brain Convolutions."

Dr. Rayner then opened a discussion on "The Treatment of Melancholia," dwelling especially on rest, outdoor exercise, feeding, and hypnotics. He said rest was indicated where there was loss of nutrition, but was harmful in many cases when combined with isolation, as in the Weir-Mitchell treatment. In some cases outdoor life, light clothing, cold baths, and regularity of meals proved most beneficial. In regard to sea voyages it should be remembered that there was an element of danger, both from the fact that the result of the change to the patient is unknown, and that there is a constant danger of suicides in melancholics. Some had said that forcible feeding need never be resorted to, but he considered it better to err on the side of over—rather than under—feeding. In his experience he had seldom found it necessary to treat the insomnia of melancholics with hypnotics, and he mentioned experiments tending rather to show that such drugs were injurious to the brain cells. Great attention should always be paid to all parts of the alimentary tract.

Dr. Fielding Blandford was sceptical about the advantage of a sea voyage, and thought that hypnotics were often very beneficial.

Professor von Benedikt on the other hand thought that often the use of narcotics made patients much worse and prolonged the disease.

Dr. Norman Kerr thought that a sea voyage with suitable companionship was often beneficial. For sleeplessness he often used the hot and cold wet pack, and as to drugs thought the best was perhaps phenacetin.

Several papers were read on the relations of various mental symptoms to certain bodily diseases.

An interesting discussion on "Insanity in Relation to Criminal Responsibility" was introduced by Dr. H. Maudsley.

### Physiology.

The work of this section was commenced by the delivery of an introductory address by Dr. Ferrier on "The Relations of Physiology and Medicine," in which he pointed out how largely recent progress had been effected by a thorough and accurate combination of the results attained by the two sciences. The difficulties met with by the clinical observer were enormous, in consequence of the complex and contradictory nature of the problems he had to unravel; yet, so soon as physiological work could be brought to bear upon them, order quickly appeared among the mass of facts which had been accumulated.

Many interesting papers were read in this section.

### Anatomy and Histology.

An address was given by the President, Mr. Henry Morris. He thought that, in its wider sense, anatomy was comprehensive enough to occupy much of the daily life of many different sets of workers; and in view of the amount of work which the student of medicine had to acquire in order to gain a diploma, he agreed with Huxley's saying that "whoever adds one tittle that is unnecessary to medical education is guilty of a very grave offence."

Professor W. Anderson opened a discussion on "Art: Its Relations to Anatomy," illustrating his remarks by lantern pictures which were taken from anatomical treatises from the time of Berengarii da Carpi to the present day. The comparative value of the different methods which have been adopted in various ages in the reproduction of anatomical pictures was considered, and the various methods of teaching anatomy were fully dealt with.

### Pathology and Bacteriology.

The presidential address was delivered by Dr. Samuel Wilks, who showed that every pathological process was accompanied by a corresponding reparative process, and that the study of the several influences exerted in the production of each was of the greatest importance. Dr. P. Manson gave a demonstration of the malaria parasite, dealing more especially with the life-history of the parasite outside the human body, showing how it was probable that the intermediate host was the mosquito. Dr. Thin offered some objections to the hypothesis of Dr. Manson, who in his reply expressed the hope that, with more complete investigations in what was really an entirely new field, these could be removed.

Many very interesting papers were read in this section.

### Ophthalmology.

The president, Mr. Power, welcomed the members of this section in a short address, and many papers of great interest were read.

### Diseases of Children.

After some remarks on the importance of the work of this section, the president, Mr. John H. Morgan, opened the discussion on congenital syphilitic manifestations in bones and joints. He discussed the pathology and clinical features of the atrophic and osteophytic varieties of the disease, and also the relation of rickets to congenital syphilis, especially in regard to the causation of cranial bosses and the occurrence of chronic effusions into joints. Many speakers joined in the discussion which followed. Mr. Rushton Parker read a paper on the treatment of hernia in children. During the last four years he had operated on 40 cases, all males, with two deaths. The chances of recurrence were very small, and Macewen's operation was the most secure, if not the easiest, method. He laid great stress on prolonged rest in the recumbent position after operation.

Mr. John Langton advocated treatment by truss only in a large majority of cases.

Professor Macewen spoke of the impossibility of dealing effectively with hernia in hospital practice by means of a truss—an opinion which was endorsed by every speaker present. In about thirty cases in which he had operated he had met with perfect success.

Dr. Ward Cousins thought the operation in children over twelve months old was eminently successful. Many speakers took part in the discussion, which was of a very interesting nature.

Dr. Handford read a paper on the "Nervous Sequelæ of Acute Infectious Diseases in Children." He inclined to think not so much that each fever was followed by its own peculiar sequelæ as that the organ affected by the virus and the distribution of the lesions accounted for the nature of the nervous sequelæ.

Among the speakers was Professor von Ranke, who said he had an impression that nervous symptoms had become more common since the introduction of the serum treatment, probably because the mortality was smaller, and more serious cases survived. Various other papers were read and discussed with much interest.

### Otology.

Sir William Dalby, in opening the section, referred to the progress made in otology since the last meeting, and showed an interesting case.

A discussion on the treatment of various forms of nerve deafness was opened by Dr. Dundas Grant.

Dr. Macnaughten Jones read a paper on "Some Etiological Considerations on the Treatment of Nerve Deafness."

Dr. Edward Law raised the question of the advantage of mercury in cases of old-standing syphilitic deafness, having found but little improvement in the hearing either from it or pilocarpin.

Mr. Field, on the other hand, thought that if suitable cases were chosen good results would be obtained from pilocarpin.

Papers were read on "Tuberculous Disease of the Middle Ear," by Dr. Milligan; on "The Treatment of Intractable Suppuration of the Middle Ear by operation through the Mastoid," by Dr. T. Barr; and on "Five Cases of Attic Disease," by Dr. Brown.

Professor Macewen opened a discussion on cerebral complications in relation to middle-ear disease, which was joined in by a large number of members, who reiterated again and again the necessity of attaining as complete a condition of asepsis as possible.

Professor Macewen, in replying, pointed out that it was absolutely the duty of the surgeon to operate in such cases with chronic aural discharge, that even in the most advanced cases complicated with serious cerebral mischief there was a chance of success, and that one was not justified in depriving the patient of that chance, however slight it might be, knowing the hopeless nature of the disease.

### Pharmacology and Therapeutics.

The main interest of this section was concentrated upon the discussion on sero-therapeutics, which was opened by Dr. Klein by a paper on "The Nature of Antitoxin." Considerable emphasis was laid on the difference between the effects produced by injecting filtered diphtheria toxin, and by inoculating with living cultures of the diphtheria bacillus; the serum from the animals treated by the first method having great power in neutralising the toxin, but only slight immunising effect, while that from animals treated in the second way had great immunising, but feeble neutralising power. Dr. Washbourne thought there were not sufficient grounds for supposing that serum injections were directly provocative of albuminuria or of anuria, as had been stated by some, both of which complications had been sufficiently common before serum-therapeutics were even thought of. Eruptions and joint pains were, however, undoubted sequelæ.

### Laryngology.

Several interesting papers were read in this section, the principal subject of discussion being the etiology of mucous polypi of the nose.

### Ethics.

The subject of advertising was introduced by Dr. Potter, who showed the numerous ways by which medical men brought discredit on their profession in this way, especially insisting that while advertising in the lower branches of the profession was properly condemned, practices differing very little in principle, perpetrated by practitioners of a higher standing, ought not to be condoned.

Several papers were read on clubs, medical aid societies, and contract practice, which excited considerable interest, and Dr. Hugh Woods introduced a discussion on hospitals and dispensaries, to which we may have to refer later.

